# Functional verification of the diphtheria toxin A gene in a recombinant system

**DOI:** 10.1186/2049-1891-3-29

**Published:** 2012-10-15

**Authors:** Jingfeng Zhang, Hengxi Wei, Xinzheng Guo, Minghua Hu, Fenglei Gao, Li Li, Shouquan Zhang

**Affiliations:** 1 Agricultural Animal Genomics and Molecular Breeding Key Lab of Guangdong Province, College of Animal Science, South China Agricultural University, Guangzhou, 510642, China

**Keywords:** Diptheria toxin A, Cell proliferation, Protein synthesis

## Abstract

Diphtheria toxin A (DTA), a segment of the diphtheria toxin (tox), inhibits protein synthesis in cells. When released from a cell, DTA is nontoxic and cannot enter other cells independently without the help of diphtheria toxin B. In this study, we artificially synthesized the *DTA* gene sequence and cloned it into pEGFP-N1 to generate the recombinant vector pEGFP-N1-DTA. This recombinant vector was then transfected into 293T cells to observe the effect of DTA protein expression on enhanced green fluorescent protein (EGFP) protein expression and the proliferation of 293T cells. After 48 h, high levels of EGFP expression were seen in control pEGFP-N1-transfected cells, whereas very low levels were seen in cells transfected with pEGFP-N1-DTA. Reverse transcription polymerase chain reaction confirmed the expression of the *DTA* gene in cells transfected with pEGFP-N1-DTA. Further, the 3-(4,5-dimethylthiazol-2-yl)-2,5-diphenyltetrazolium bromide (MTT) assay revealed a significant difference in cell proliferation between the control group and the pEGFP-N1-DTA-transfected group. Using the expression of EGFP expression as an indicator, this study revealed that DTA expression can inhibit intracellular protein synthesis and cell proliferation.

## Introduction

Diphtheria toxin, a 58-kDa exotoxin secreted by *Corynebacterium diphtheriae* transduced by a beta bacteriophage, can inactivate the ADP-ribosylation of elongation factor 2 (EF2), resulting in the inhibition of protein synthesis and cell death. Diphtheria toxin can be cleaved by trypsin into fragment A (193 aa) and fragment B (342 aa). Diphtheria toxin fragment A (DTA) is a 21-kDa protein that is responsible for the inactivation of EF2, causing inhibition of protein synthesis. Fragment B is a 37-kDa protein that can bind to cell-surface receptors and deliver DTA into cells [1,2]. Studies have shown that the toxicity of the DTA protein is very high; a molecule of DTA is able to cause cell death is non-toxic in the extracellular form [3,4]. Because of its toxicity and specific molecular structure, DTA has been widely used to kill specific cells in targeted tumor treatment, such as for human breast cancer and lung cancer [5-10]. The current study provides a new strategy to directly and indirectly verify the effect of the DTA protein on intracellular protein synthesis and cell proliferation.

## Materials and methods

### Materials

The pEGFP-N1 vector (Clontech) and 293T cells were available in our laboratory. The KOD FX polymerase was obtained from Toyobo. T4 DNA ligase, restriction enzymes, and DL2000 were obtained from Takara. Agarose and kits related to DNA were purchased from OMEGA. 3-(4,5-Dimethyl-2-thiazoly) 2,5-diphenyl-2-tetrazolium bromide (MTT) was purchased from Fluka. Dulbecco’s modified Eagle medium (DMEM/F12 [Invitrogen]), fetal bovine serum (FBS; Hyclone), Lipofectamine 2000, and the reverse transcription polymerase chain reaction (RT-PCR) kit were purchased from Invitrogen. Other reagents were all pure preparations of analytical grade.

### DTA gene sequence

The DTA amino acid sequence includes residues 1–193 of the diphtheria toxin. Therefore, the coding sequence of diphtheria toxin (GenBank accession no.: 2650491) was obtained from the National Center for Biotechnology Information (NCBI) and first 600 bp was synthesized by Qinglanbio [11]. The *DTA* sequence was cloned into the pUC-19 vector and verified by nucleotide sequencing by Sangon Biotech (Shanghai) using forward (5'-ATGGTGAGCAGAAAACTGTTTGCGTC-3') and reverse (5'-TTGGCCACGTTTTCCACGG-3') primers.

### Plasmid construction

The pEGFP-N1-DTA plasmid was obtained by polymerase chain reaction (PCR) using pUC-19-DTA as a template and the BglII-DTA forward (5'-GAAGATCTCATGGTGAGCAGAAAACTGTTTGCGTC-3') and the EcoRI-DTA reverse (5'-GGAATTCGTTGGCCACGTTTTCCACGG-3') primers, to obtain DTA cDNA and insert it into the multiple cloning site (MCS) of the pEGFP-N1 plasmid. The resulting pEGFP-N1-DTA plasmid contained *DTA* in the same open reading frame (ORF) as EGFP and expressed the DTA-enhanced green fluorescent protein (EGFP) fusion protein under the control of the cytomegalovirus (CMV) promoter. For the PCR, the reaction mixture was heated to 94°C for an initial 5 min and then amplified by denaturing at 94°C for 30 s, annealing at 60°C for 30 s, and extending at 72°C for 1 min, for a total of 35 cycles, with a final extension at 72°C for 10 min.

### Cell transfection

The 293T cells were maintained in DMEM/F12 medium supplemented with 10% fetal bovine serum, and 100 U/mL penicillin/streptomycin, and cultured in 37°C and 5% CO_2_. When the cell cultures achieved >85% confluence, the 293T cells were removed by trypsinization treatment, washed in phosphate-buffered saline (PBS). The 293T cells were cultured in 24-well cell plates at a density of 5.0 × 10^4^ cells/well, and at approximately 75% confluence, cells were transfected with plasmids (1 μg/well) using Lipofectamine 2000 (2 μL/well). After 24 and 48 h, the expression of EGFP was detected by fluorescence microscopy.

### RT-PCR

Cells from the three groups (experimental, positive control, and negative control) were collected 48 h post-transfection, and total RNA was extracted. RNA isolation and RT-PCR were performed according to the manufacturers’ instructions.

### Western blot analysis

First, 293T cells were transfected with pEGFP-N1 in the control group and pEGFP-N1-DTA in the experimental group. The same numbers of cells were collected respectively from these two groups 48 h post-transfection. Second, cells from the two groups were lysed respectively in cell lysis buffer (P0013, Beyotime) supplemented with phenylmethanesulfonyl fluoride for 15 min. Lysed cells were centrifuged at 4°C, 14000×g supernatants were collected for western blotting using 15 μL protein supernatants and subjected sodium dodecyl-polyacrylamide gel electrophoresis (SDS-PAGE) and transferred to an immobilion-p transfer membrane (Millipore) and blocked with 5% non-fat milk in tris-buffered saline containing tween20 (TBST; 50 mM Tris base, pH 7.5, 150 mM NaCl, and 0.1%(w/v) Tween 20). The membranes were incubated with affinity-purified GFP-tag antibodies (1:4000 dilution, Abmayt) at 4°C overnight. The membranes were washed three times with TBST and incubated with goat anti-mouse IgG (1:2000) for 1–2 h at room temperature. Bands were visualized using the ECL western blotting analysis system (Proteinsimple). The membranes were washed with antibody eluent (glycine, 0.025M, sodium dodecyl sulfate, 0.05M, pH 2.0), and treated with anti-β-actin antibody (1:2000, Abmayt).

### MTT assay

The 293T cells cultured in 96-well cell plates were transfected and incubated for 24 or 48 h. Supernatants were removed, 100 μL DMEM and 20 μL MTT (5 mg/mL) were added in the absence of light to each well, and the cells were further incubated. After 4 h, the 96-well plate was centrifuged at 20°C and 425×g. The supernatant was then removed using filter paper, 150 μL dimethylsulfoxide (DMSO) was added to each well, and the plate was shaken at 80 rpm for 15 min. Subsequently, the absorbance at 490 nm was measured for each well, including blanks, using a microplate reader.

### DTA toxicity assay

Cells were transfected, as previously described, with pEGFP-N1-DTA in the experimental group or pEGFP-N1 for the control group. On the third and fifth days post-transfection, the 293T cells were seeded into 12- and 6-well plates, respectively, and EGFP expression and cell proliferation were assayed.

### Statistics analysis

In MTT assay experiment, each group had three wells, and the date value of each group was the average one of three wells. Data was processed by SAS 8.1 (SAS Institute Inc., Cary, NC) statistical software. Significance was declared at P ≤ 0.05.

## Results

### Construction of pEGFP-N1-DTA plasmid

Under ultraviolet light, EGFP protein emits green fluorescence that can be directly observed. Therefore, inhibition of EGFP protein expression by DTA can be easily and directly verified. In the pEGFP-N1 vector, the MCS is located between the CMV promoter and the *EGFP* coding sequence, and genes without stop codons cloned into the MCS will be expressed as fusion proteins with the N-terminus of EGFP if they are in the same reading frame as EGFP. We cloned the DTA coding sequence into the MCS in the same ORF as EGFP, generating a plasmid, pEGFP-N1-DTA.

**Figure 1:**
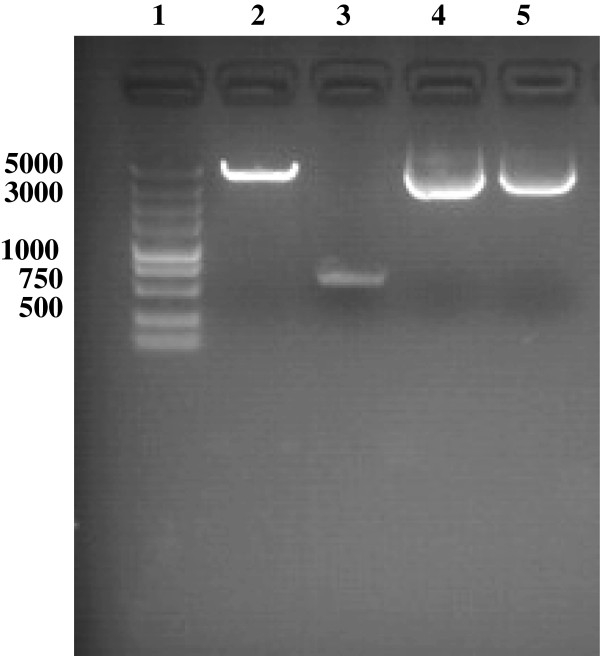
**Electrophoretic analysis of the pEGFP**-**N1**-**DTA construct.** Lane 1, DNA marker (5 kb); lane 2, pEGFP-N1 sequence after digestion with BglII and EcoRI ; lane 3, DTA sequence after digestion with BglII and EcoRI; lane 4, pEGFP-N1 plasmid; lane 5, pEGFP-N1-DTA plasmid.

**Figure 2:**
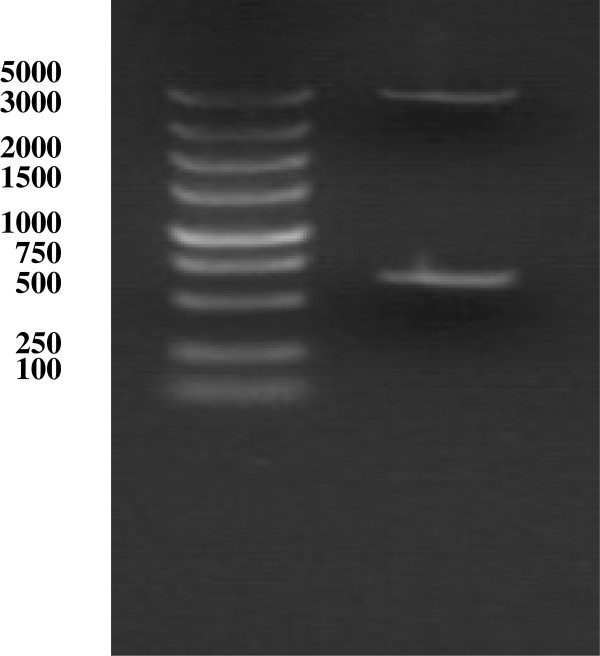
**Electrophoretic analysis of pEGFP**-**N1**-**DTA digested with two enzymes with BglII and EcoRI.** Lane 1, DNA marker (5 kb); lane 2, pEGFP-N1 fragment (top band) and DTA fragment (bottom band).

**Figure 3:**
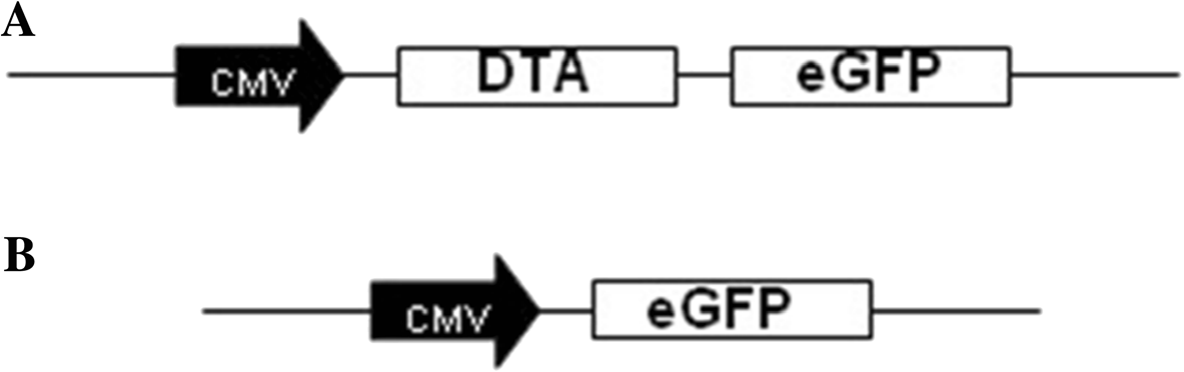
**Plasmid vector maps of pEGFP**-**N1 and pEGFP**-**N1**-**DTA.A**) Main elements of pEGFP-N1-DTA. The CMV promoter drives the expression of the *DTA* coding sequence and *EGFP* coding sequences in the same ORF. **B**) Main elements of pEGFP-N1. The CMV promoter drives *EGFP* gene expression only.

Figure 1 shows the electrophoretic separation of restriction enzyme (BglII and EcoRI)-digested DTA (600 bp) and pEGFP-N1 plasmid, the original pEGFP-N1 plasmid, and the pEGFP-N1-DTA plasmid. Figure 2 shows the electrophoretic separation of the DTA and parent pEGFP-N1 fragments following digestion of the new construct by BglII and EcoRI. These results verify that the pEGFP-N1-DTA vector (Figure 3) has been successfully constructed.

### EGFP expression assay and RT-PCR

**Figure 4:**
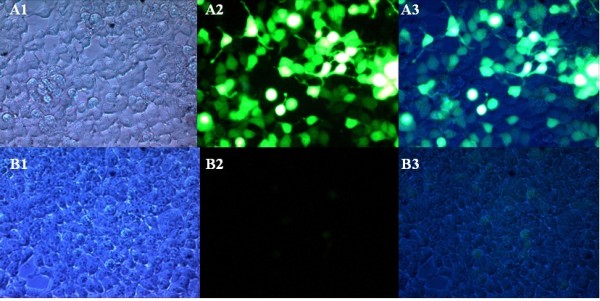
**Transfection of the pEGFP**-**N1 vector and the pEGFP**-**N1**-**DTA vector into 293T cells.A**) Transfection of pEGFP-N1 into 293T cells: high EGFP expression at 48 h post-transfection. **B**) Transfection of pEGFP-N1-DTA into 293T cells: low levels of EGFP expression. **A1** and **B1**, pictures taken under white light; **A2** and **B2**, pictures taken under UV light; **A3** and **B3**, merged pictures. Magnification, 400×.

**Figure 5:**
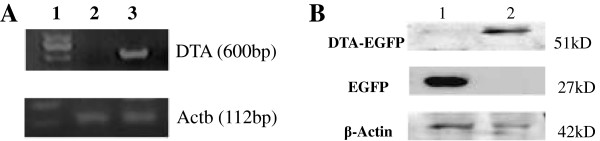
**RT**-**PCR for transcription of genes.A**) Agarose gel electrophoresis analysis of RT-PCR amplicons. Lane 1, DNA marker; lane 2, cells transfected with pEGFP-N1; 3, cell transfected with pEGFP-N1-DTA; expression of DTA was seen only in the second group, and not in the first. Actb was used as an internal control. **B**) Western blot analysis of cell extracts using anti-GFP and anti-β-actin antibodies. Lane 1, control group transfected with pEGFP-N1; lane 2, experimental group transfected with pEGFP-N1-DTA. EGFP protein was detected in control group, and the DTA-EGFP fusion protein in experimental group. The expression level of DTA-EGFP fusion protein was lower than EGFP, and the expression of β-actin in experimental group was less than that in control group.

Three conditions were tested in this experiment. The experimental group and the positive control group consisted of 293T cells transfected with pEGFP-N1-DTA and pEGFP-N1, respectively, and the third group was the negative control, which was incubated with complete medium alone. Fluorescence microscopy showed high expression levels of the EGFP protein in positive control 293T cells at 48 h post-transfection; however, very low-level expression was observed in the experimental group (Figure 3). No expression of EGFP was observed in the negative control group (Figure 4). Figure 5 shows that RT-PCR detected DTA expression in only the experimental group and not in the positive and negative control groups. Western blot analysis confirmed that the expression level of DTA-EGFP fusion protein in the experimental group was lower than EGFP in positive control. Moreover, the expression of β-actin in the experimental group was also less than that found in the positive control group. These data show that the expression of DTA inhibits the expression of EGFP, confirming the effect of DTA.

### Cell proliferation assay

**Figure 6:**
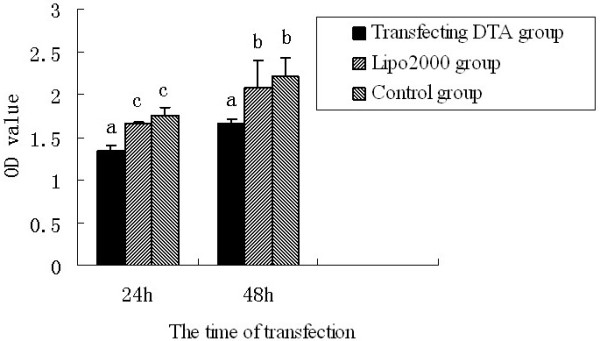
**Cell proliferation assay.** The optical density at 490 nm is represented on the y-axis and the time after transfection on the x-axis. Bars labeled with the same letter did not show significant differences (p > 0.05); bars labeled with different and successive letters differed significantly (0.01 < p < 0.05); bars labeled with different letters that are not successive differed more significantly (p < 0.01).

Since DTA has been previously shown to inactivate the ADP-ribosylation of EF2, it is likely to play a negative role in cell proliferation. This study was carried out to determine the effect of DTA expression on cell proliferation. The 293T cells, however, grow very fast, and splitting transfected cells into a new plate will lead to inaccurate data, thus, we assayed cell proliferation at 24 and 48 h after transfection. Three treatments were analyzed in this experiment: the experimental group, in which the 293T cells were transfected with the pEGFP-N1-DTA plasmid; the negative control group, that were transfected with cell culture medium alone; and the positive control group, in which cells were transfected with pEFGP-N1. Figure 6 shows that at 24 h post-transfection, the fluorescence intensity was significant different between the transfected and negative control groups (P < 0.01). At 48 h post-transfection, the fluorescence intensity between the transfected group and the negative and control groups were significantly different (p < 0.05). On the other hand, the differences between the negative and control groups at 24 and 48 h were not statistically significant (p > 0.05) indicating that the Lipofectamine 2000 had no effect on our experimental observations. This experiment showed that the DTA protein is toxic to cells and significantly affects cell proliferation.

### Toxicity of DTA

**Figure 7:**
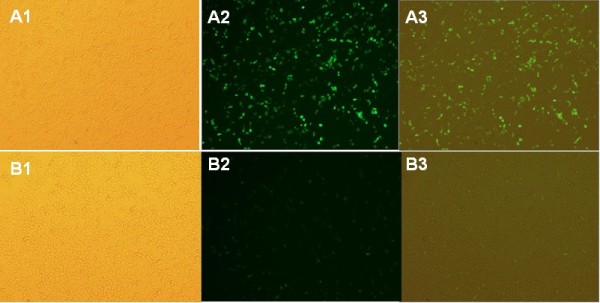
**Transfection of 293T cells with pEGFP**-**N1 and pEGFP**-**N1**-**DTA vectors for 24 h.A**) High EGFP expression was seen for pEGFP-N1-transfected 293T cells. **B**) Lower EGFP expression levels was seen for pEGFP-N1-DTA-transfected cells at 24 h post-transfection; **A1** and **B1**, images taken under white light; **A2** and **B2**, images taken under UV light; **A3** and **B3**, merged pictures. Magnification, 200×.

**Figure 8:**
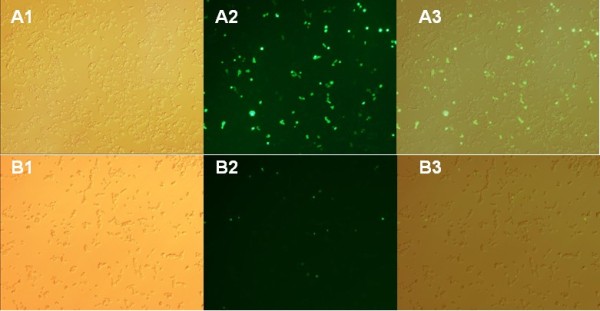
**Transfection of 293T cells with pEGFP**-**N1 vector(A)and pEGFP**-**N1**-**DTA vector(B)at six days post**-**transfection.** Few cells emitting green light were observed in the group transfected with pEGFP-N1-DTA, whereas many were seen in the control group; the cell density in the second group was less than that in the first group; **A1** and **B1**, images taken under white light; **A2** and **B2**, images taken under UV light; **A3** and **B3**, merged pictures. Magnification, 200×.

To further investigate the cytotoxicity of the DTA protein, we first cultured 293T cells in 24-well plates and were then transfected with pEGFP-N1-DTA or pEGFP-N1. The cells were then cultured in 12-well plates on day 3 post-transfection, and in 6-well plates on day 5 post-transfection. Consistent with our previous observations, Figure 7 shows that at 24 h post-transfection, the expression level of EGFP was much lower in cells transfected with pEGFP-N1-DTA than in control cells. Figure 8 shows that at 6 days post-transfection, the number of cells expressing EGFP was low in the group transfected with pEGFP-N1-DT but larger in the control group. In addition, the cell density was also lower in the former group than the latter. This experiment further confirmed the toxicity of the DTA protein to cells and its ability to inhibit cell proliferation.

## Discussion

The toxicity of the DTA protein is very high, where a single molecule is able to cause cell death [3]. Because of its high toxicity, DTA has been widely used in targeted tumor treatment and for generating animal models. Our data show that the DTA protein can inhibit intracellular protein expression and is toxic to cells, thereby affecting cell proliferation. Because *C*. *diphtheriae* can infect humans, we synthesized the *DTA* coding sequence by artificial synthesis rather than from the bacterium.

Under excitation by UV light, EGFP emits visible green fluorescence. Therefore, our experimental design involved using the DTA protein to inhibit EGFP protein synthesis in order to directly verify DTA protein function. Consistent with our hypothesis, our results showed that after transfecting 293T cells with pEGFP-N1-DTA, the level of EGFP expression was very low compared with EGFP expression in control cells. Moreover, the MTT assay was used to investigate the effect of DTA on cell proliferation. Since Lipofectamine 2000 is toxic to cells, we added a Lipo2000 control group to this experiment. The present study showed that the difference in cell proliferation between the Lipo2000 group and the control group was not significant, demonstrating the negligible effect of Lipofectamine 2000 on proliferation. Further, the difference between cell survival in the experimental group and the two control groups at 48 h post-transfection was significant, but not more significant than that seen at 24 h.

In the DTA toxicity assay, we initially cultured 293T cells in 24-well plates and then transfected with pEGFP-N1-DTA or pEGFP-N1. The 293T cells proliferate very fast and need sufficient space to grow; additionally, the expression of EGFP may cause death in a small number of cells, which will affect the growth of other cells. Therefore, when 293T cells reached 85% confluence, the cells were split into 12-well plates and then 6-well plates on the third and fifth day post-transfection, respectively. On the sixth day, we found that the density of the cells in the experimental group was less than that found in the control group, confirming that the DTA protein inhibited 293T cell proliferation. Moreover, few cells were found to express EGFP protein in the experimental group, whereas many cells produced EGFP in the control group. This experiment further demonstrated the toxicity of the DTA protein and its inhibitory effect on cell growth.

Based on its function and characteristics, DTA has been widely used in tumor treatment, gene functional studies, and in the generation of animal models, among other applications [5-10]. In tumor treatment, the *DTA* gene was cloned into the tetracycline recombinant viral vector and A549 cells were inoculated subcutaneously into nude mice. When the A549 tumor grew to 352 mg, viral supernatant containing the *DTA* gene was injected into the tumor resulting in resolution of the tumor [5]. With regard to gene functional studies and the generation of animal models, the lacZ coding sequence containing loxP at both terminals was inserted into the DTA coding sequence downstream of the ATG codon. The floxed lacZ-DTA sequence was then targeted into the location of the ROSA26 gene to generate a R26:LacZ/DTA mouse model, which has been used to delete specific cells *in vivo*, such as pyramidal neurons, oligodendrocytes, B cells, and hepatocytes [7]. Researchers have attempted to verify the function of cardiac muscle cells in cardiac arrhythmia using myosin heavy chain α (MHCα) to promote *DTA* gene expression and created a transgenic mouse model [12]. In all, the *DTA* gene has been widely used, and the results the current study can provide a basis for future research. In 2009, Wu *et al*. [13] discovered stem cells in the adult ovary, which was a challenge contrary to existing knowledge. However, there are still many scientists who do not accept this because when these stem cells are replaced in mouse ovaries treated with busulfan, not all of the recipient mice were able to give birth to offspring. Researchers can now make use of the *DTA* gene to specifically delete oocytes in mice, and use this mouse model as the recipient without the need for chemical treatment, and the resulting birth rate may provide a convincing answer to resolve the argument of whether the cells from the ovary are stem cells.

This study has directly verified the effect of the DTA gene on intracellular protein synthesis and cell proliferation, and can be regarded as the basis for subsequent research. It is believed that the DTA gene will be applied to future research studies because of these effects. In addition, the toxicity of the DTA protein to cancer cells may be not only due to inhibition of protein synthesis, but may also arise because of the induction of cell apoptosis [5,14]. However, this problem is outside the scope of the present study and will be investigated in future studies.

## Competing interests

The authors declare no conflict of interest.

## Authors’ contribution

JZ carried out the molecular genetic studies and drafted the manuscript. SZ advised JZ in performing the study and writing the manuscript for publication. And HW, XG, MH, FG and LL gave much help in this experiment. All authors read and approved the final manuscript.
